# Finger orthoses for management of joint hypermobility disorders: Relative effects on hand function and cognitive load

**DOI:** 10.1177/0309364620956866

**Published:** 2020-09

**Authors:** Anne-Mette Jensen, Joan Quist Andersen, Lena Quisth, Nerrolyn Ramstrand

**Affiliations:** 1 Ortos A/S, Odense, Denmark; 2 Ortos A/S, Kolding, Denmark; 3 CHILD Research Group, School of Health and Welfare, Jönköping University, Jönköping, Sweden

**Keywords:** fNIRS, silver splint, Ehlers-Danlos syndrome, hand orthosis, hand function

## Abstract

Joint hypermobility refers to joints that move beyond their normal limits. Individuals with hypermobility of the fingers experience difficulties in activities of daily living. Finger orthoses are available for managing hypermobility of the fingers, but their effectiveness has received little attention in scholarly literature.

**Objectives:**

To determine if use of custom fit finger orthoses leads to improvements in time needed to perform standardised hand function tests, and attentional demand required to perform these tests, in individuals with joint hypermobility syndrome, Hypermobile Ehlers-Danlos syndrome or Classical Ehlers-Danlos syndrome.

**Study design:**

Repeated-measures study.

**Methods:**

Fourteen participants performed three different hand function tests (target box and block test, writing and picking up coins), with and without their finger orthoses. Time to complete each test was recorded as a measure of functional performance. Brain activity was recorded in the pre-frontal cortices as a measure of attentional demand.

**Results:**

Functional performance significantly improved for all but one test (picking up coins with non-dominant hand) when participants wore finger orthoses (p < 0.05). Activity in the pre-frontal cortex was lower when using the orthosis to perform the coin test (dominant hand; p < 0.05). No differences were observed in other tests (p > 0.05).

**Conclusions:**

Results suggested that finger orthoses improved hand function and provided limited evidence to suggest that they may also affect attentional demand. While the limited sample does not provide conclusive evidence supporting the use of finger orthosis in this clinical population, results warrant further investigation in large scale longitudinal studies or randomised controlled trials.

## Background

Joint hypermobility is defined as movement of joints beyond their normal range of motion, and is typically caused by a collagen deficiency. When joint hypermobility occurs in combination with other symptoms and affects an individual’s daily life, it is often diagnosed as joint hypermobility syndrome (JHS). The prevalence of JHS has been estimated as high as 2-3/100.^1^ The presence of joint hypermobility in combination with Ehlers-Danlos syndrome (EDS) is a distinct clinical diagnosis. There are 13 subtypes of EDS,^2^ with Hypermobile Ehlers-Danlos syndrome (hEDS) and Classical EDS (cEDS) being two of the most common types.^3^ Joint hypermobility is listed within the diagnostic criteria for both of these types of EDS.^2^

Although diagnoses of JHS, hEDS and cEDS are based upon different sets of diagnostic criteria, there is a great deal of overlap between the conditions and it has been suggested that they represent the same clinical entity.^4,5^ All of these diagnoses are associated with functional and psychosocial impairments.^6^ Major symptoms include hypermobility, chronic fatigue, impaired sleep, anxiety and depression.^7^ Most people also experience constant pain.^1,4^ Pain is most often related to injuries, growth, sports and repetitive task such as handwriting.^1,8^ Forty per-cent of young students with hypermobility report difficulties with their handwriting, and 48% are considered to be clumsy.^9^

Individuals with hypermobility disorders report that they need to be constantly aware of the placement of their hands and feet.^10^ This is likely a consequence of muscle weakness combined with reduced proprioceptive acuity,^11–14^ and can result in a need to allocate more attentional demand to performance of motor activities. The need for persons with disabilities to invest more attention and effort in performing motor tasks has received much attention in recent years.^15–18^ Attentional demand is associated with increased brain activation in the pre-frontal cortex^15,18^ and can be measured in dynamic situations using either electroencephalography (EEG) or Functional Near-Infrared Spectroscopy (fNIRS). While EEG measures voltage fluctuations resulting from electrical activity in the brain,^19^ fNIRS measures relative changes in oxygenated and deoxygenated haemoglobin.^20^

Management of hypermobility in the hands and fingers of individuals with JHS, hEDS or cEDS typically involves physiotherapy or occupational therapy interventions. These include, but are not limited to, proprioceptive-based exercises and targeted exercise-based interventions.^21,22^ Custom fit finger orthoses are commonly prescribed to manage finger hypermobility of clients with EDS in Denmark, and the Ehlers-Danlos Society in Denmark estimates that more than 40% of members use finger orthoses made of silver. There is, however, very limited research on their use and potential benefits or disadvantages.^21^

Finger orthoses for management of joint hypermobility are typically fabricated from metal or plastic and made to order based on measurements or casts provided by an orthotist. They may vary in the specifics of their design, but their general aim is to facilitate hand function and minimise pain by stabilising joints and limiting joint range of motion. There is also some suggestion that orthoses can facilitate proprioception,^23^ although the authors are unaware of studies specifically investigating finger orthoses. A 2013 systematic review identified only one study, with poor methodological quality, investigating orthotic management for individuals with hypermobility.^21^ That study investigated use of a wrist hand orthosis on four students with JHS and suggested that the device was not effective in reducing pain or increasing writing speed.^24^

The aim of the present study was to determine if use of custom fit finger orthoses can reduce the time required to perform tests of hand function, and determine whether they can reduce the attentional demand required to perform hand function tests in individuals with hypermobility disorders.

To the authors’ knowledge, this study represents the first of its kind to assess the potential benefits of finger orthoses for individuals with JHS, hEDS or cEDS. It also represents the first study in which attentional demand has been objectively measured in this group of individuals.

We hypothesised that:

- Time to perform functional hand tests would be significantly reduced in individuals with hypermobility disorders when they were using finger orthoses compared to no finger orthoses.
- Activity in the prefrontal cortex would be significantly reduced when individuals with hypermobility disorders performed functional hand tests while using finger orthoses compared to no finger orthoses.

## Methods

A repeated-measures study was undertaken in which participants diagnosed with generalised hypermobility were tested with and without their prescribed finger orthoses. Participants were tested with and without the orthosis in a single test session. All testing was conducted in a quiet room within one of three orthotic clinics in Denmark (Ortos Odense, Ortos Kolding and Ortos Aarhus). Reporting of the research has been conducted in accordance with the STROBE Statement.^25^

### Participants

Participants in this study were a convenience sample of adults diagnosed with JHS, hEDS or cEDS, who were registered as clients at Ortos orthotic clinics in Denmark. All participants were female as cEDS and hEDS have been found to affect women to a much greater extent than men.^26^ They were invited to take part in the study if they had been prescribed and fitted with a silver finger orthosis over metacarpal phalangeal joint 1 (MCP-1) and at least one interphalangeal joint (IP) (Hand M Aid, Asker, Norway/ Silver Ring Splint company, Charlottesville, VA).

Participants were required to be between the age of 18 and 65 and able to understand spoken and written Danish. Clients with cognitive impairments who could not understand the aims and procedures of the study were excluded. Prior to testing, participants were informed of the study procedures and provided written informed consent.

### Descriptive variables

Descriptive variables were collected at initiation of the study. These data included the age of the participant, handedness, occupation, level of pain in daily life (measured on a scale of 0–10), type of orthoses used and duration of use.

### Independent variables

Finger orthoses served as the independent variable. Photographs of the hands of all participants were taken to document the number and design of finger orthoses used by each participant and the specific joints being stabilised.

### Dependent variables

The major dependent variables of interest were the time to complete hand function tests and cortical brain activity in the left and right dorsolateral pre-frontal cortex (DLPFC). As both dominant and non-dominant hands are affected by hypermobility, and attentional demand may increase when performing activities with the non-dominant hand, we chose to test both hands.

Three specific tests were performed by each participant. Each test was designed to measure different aspects of hand function. All tests were performed with the participant seated comfortably at a table. Each test required use of different grips and is described in detail below. Time to complete each test was recorded with a stopwatch.

Brain activity was recorded using an fNIRS system, incorporating 8 detector optodes and 8 illumination optodes, resulting in a total of 20 specific channels (NIRSport, NIRx Medical Technologies). fNIRS was selected over EEG as it has better spatial acuity with effects being able to be localised within 1–2 cm of the area of the brain that is activated.^27^ fNIRS provides an indication of cortical brain activity by measuring the relative concentration of oxygenated haemoglobin (HbO_2_) in specific regions of the brain. This is achieved by measuring the absorption of light within the near-infrared spectrum (650–1000 nm). Changes in the relative concentrations of HbO_2_ cause predictable changes in the intensity of reflected light and can be quantified according to the Beer-Lambert law.^28^ A typical haemodynamic response to a stimulus would be a localised increase in blood flow to the activated region of the brain and a subsequent increase in the concentration of oxygenated haemoglobin.^29^

#### Targeted box and block test

The Box and Block test is considered a gold standard measure of gross manual dexterity.^30^ When performing the original version of the test participants are required to move as many 2.5 cm^2^ blocks as possible over a 12.2 cm partition in 1 min. The targeted Box and Block test (tBBT) is a modification of this test^31^ in which placement and order of block pick-up is standardised, reducing variability in the trajectory employed to move blocks over the partition. This allows for comparison within and between participants. In this study, the tBBT was repeated twice with the dominant hand, and twice with the non-dominant hand. This was of interest as it was expected that performance on tests would be slower with the non-dominant hand and would require greater attentional demand. Tests were repeated with and without the finger orthoses.

#### Writing test

Handwriting is difficult for many people with hypermobility. In this study, we chose to evaluate participants’ ability to write with a pen using the writing subtest from the Jebsen Taylor Hand Function Test.^32^ This test required participants to read sentences that were presented to them on a slip of paper and to write the sentence out themselves using a ball-point-pen. The validated English version of the test uses sentences representing a third grade reading level and includes all 24 letters of the alphabet. In the absence of a Danish version of the test, we consulted with a primary school teacher to select several Danish sentences that were representative of a grade 3 level of reading. Each sentence contained the same number of words and length of words. Sentences presented to participants were randomly selected each time the test was performed with no single sentence being presented more than once to the same person. Participants performed the test twice with the finger orthoses and twice without finger orthoses. Only the dominant hand was tested as participants found it too difficult to write with their non-dominant hand.

#### Picking up coins test

**Figure 1.**
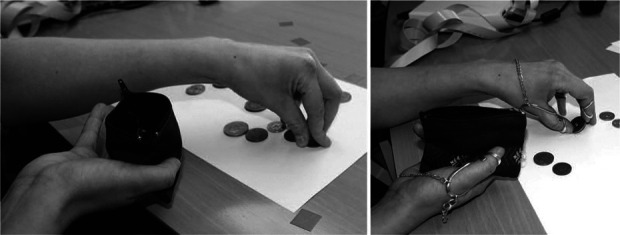
Participants performing the picking up coins test with and without finger orthoses.

The picking up coins test is a subtest in the Sollerman test^33^ and is used to evaluate pinch grip function. The original test requires the person performing the test to pick up four coins of different sizes from a flat surface and to put them in a purse mounted on a wall. As this test is quite short in duration, and it has been recommended that fNIRS trial duration should be between 20 s and 1 min^34^ to account for the lag in haemodynamic responses and oscillations of arterial blood pressure, we extended the length of the test. In our modified version, participants were required to pick up 18 coins of different sizes from a table and place them in a small purse which they held in their hand. Participants repeated the test twice, first with the dominant hand, then the non-dominant hand. This was repeated with and without the finger orthoses. Figure 1 includes photographs of a participant performing the coin test with and without finger orthoses.

### Procedure

**Figure 2.**
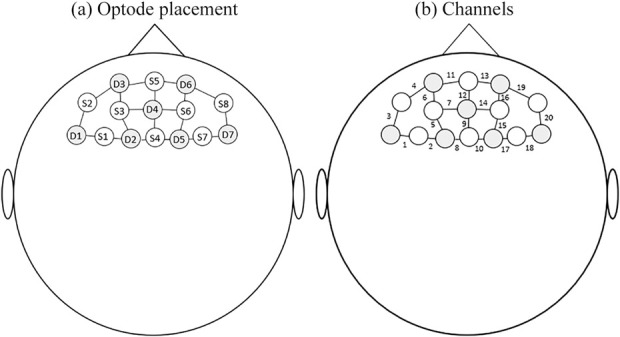
(a) Positioning of optodes; S = source D = detector. (b) Data channels created between optodes.

Testing was conducted on a single occasion with and without the participants’ own finger orthoses. For practical reasons, testing began with the tBBT followed by the writing test, and finally the coin test. Due to the time required to set up each test, it was considered impractical to randomise the order of the tests. The order of trials within each test (i.e. with or without orthoses) was randomised to counterbalance the conditions and account for potential order effects. Prior to performing tests of hand function, participants were fitted with the fNIRS system. Optodes were positioned according to the international 10–20 system for electrode placement and specifically positioned to cover Brodmann’s areas 9 and 46.^35^ These areas were selected as they are considered part of the DLPFC which has been associated with attentional demand and mental effort in previous studies that have used fNIRS to record cortical brain activity.^36,37^ Figure 2 presents the topographical layout used. The same investigator (NR) was responsible for fitting the fNIRS measurement caps and positioning optodes. Each illumination optode emitted infrared light at wavelengths of 760 and 850 nm and a frequency of 7.81 Hz.

After each specific test was explained to participants, baseline fNIRS signals were collected for a 30 second period. During this time, the participant was requested to remain seated, without moving or talking. Participants were signalled to start the test after initial baseline values were recorded. One investigator used a digital trigger to mark the fNIRS file at the beginning and end of each baseline period, and at the beginning and end of each trial. Separate fNIRS data files were created for each test of hand function, always beginning with a baseline measurement. A second investigator timed the duration of each trial using a stopwatch.

### Control of potential bias

The researchers were experienced in the procedures, including the use of fNIRS equipment. To standardise procedures, the same investigator was responsible for providing instructions to participants while another was always responsible for fitting the fNIRS measurement caps and positioning optodes.

### Data processing

fNIRS data were pre-processed using nirsLAB software (NIRx Medical Technologies, Los Angeles, CA, USA). Channels were removed if they had a coefficient of variation > 15%.^38^ The coefficient of variation, defined as the standard deviation divided by the mean value, is a measure of the signal-to-noise performance of each channel and represents a metric of signal quality. In the absence of a consensus on an appropriate threshold for the coefficient of variation, a cutoff of 15%, the maximum recommended value by the manufacturer, was selected. This value was selected considering that subjects were moving their arms during testing and this may cause occasional spikes affecting the standard deviation. Signals were bandpass filtered with a low cutoff frequency of 0.01 Hz and a high cutoff of 0.2 Hz. This served to remove low-frequency oscillations caused by heartbeat, breathing and drift.^39^ Haemodynamic states were calculated in nirsLAB software using the modified Beer-Lambert Law. Differential pathlength factors for each wavelength emitted by the optodes (760 and 850 nm) were set at 7.25 (760 nm) and 6.38 (850 nm) while molar extinction coefficients for oxyHb were 1486 (760 nm) and 2526 (850 nm) and 3843 (760 nm) and 1798 (850 nm) for de-oxyHb, measured in units of (1/cm)/(moles/litre).^40^ Relative changes in oxyHb and de-oxyHb were normalised relative to each individuals’ baseline values.

Data were modelled for the duration of each trial, determined using the digital trigger points that had been marked in the file. A region of interest (ROI) representing the left DLPFC was generated by averaging channels 4, 5, 6 and 7, while a ROI representing the right DLPFC was generated by averaging channels 14, 15, 16 and 19 (see Figure 1). OxyHb and de-oxyHb data for each trial were block averaged for each condition (box and block, writing and coin tests) using averaged signals from each ROI. Outliers were removed if they exceeded 1.5 x interquartile range.

### Statistical methods

As data were not normally distributed (Shapiro-Wilk < 0.05) non-parametric tests were used for all statistical analyses. Time to perform each trial was averaged for each individual within each condition and paired data for each participant was compared using a Wilcoxon signed-rank test. Given that data were non-parametric, effect sizes were calculated as *r* = Z/√N (where N = number of observations rather that the number of pairs of observations.^41^ Effect sizes were interpreted in accordance with recommendations of Cohen (small ≥ 0.1, medium ≥ 0.3, large ≥ 0.5).^42^

fNIRS data were analysed separately for the left and right DLPFC. Wilcoxon signed-rank tests were used to establish if significant differences existed between average OxyHB activity for the orthosis and no orthosis conditions. Graphs of average OxyHb were used to demonstrate cortical brain activity over time. The *r* statistic was again used to represent effect size. Spearman’s rho was used to determine the relationship between task performance and cortical brain activity. p-values were set at 0.05.

## Results

### Participants/descriptive data

**Figure 3.**
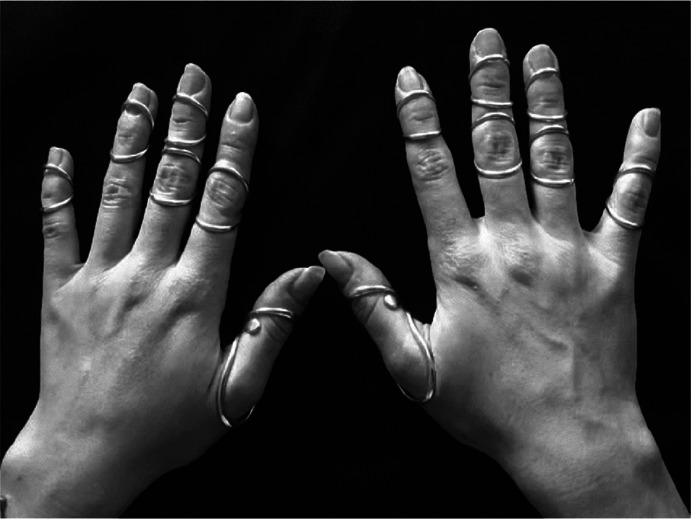
Participant 3 wearing her finger orthoses.

In all, 14 females agreed to participate. Patient characteristics are presented in Table 1. Average age of participants was 39.8 years (range: 19–65) and most had been wearing finger orthoses for more than a year. Three participants had worn their orthoses for less than 6 months. Six had taken early retirement due to their disabilities. All but two were right hand dominant. Participants wore an average of 10 finger orthoses (range: 6–18) including at least one orthosis stabilising MCP-1. Figure 3 is a photograph of participant 3 wearing her finger orthoses. All participants reported pain at rest (mean = 3.9; SD = 1.6) and when performing repetitive tasks with their hands (mean = 6.6; SD = 2.1).

**Table 1.**
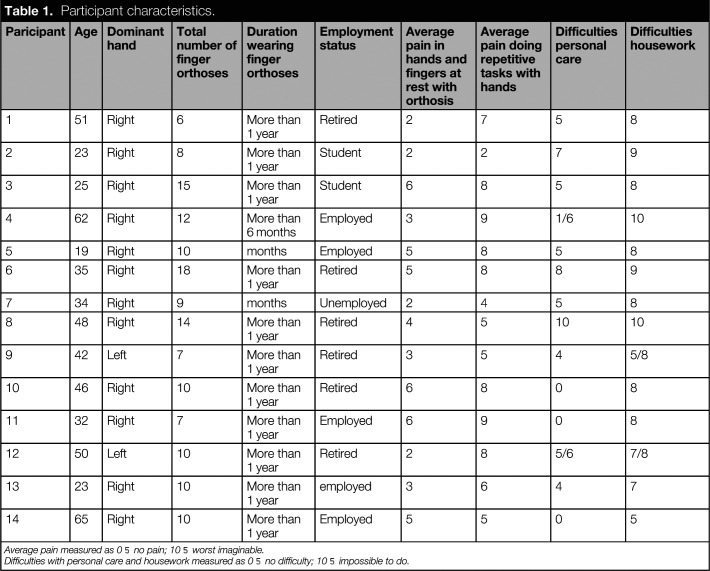
Participant characteristics.

Paricipant	Age	Dominant hand	Total number of finger orthoses	Duration wearing finger orthoses	Employment status	Average pain in hands and fingers at rest with orthosis	Average pain doing repetitive tasks with hands	Difficulties personal care	Difficulties housework
1	51	Right	6	More than 1 year	Retired	2	7	5	8
2	23	Right	8	More than 1 year	Student	2	2	7	9
3	25	Right	15	More than 1 year	Student	6	8	5	8
4	62	Right	12	More than 6 months	Employed	3	9	1/6	10
5	19	Right	10	months	Employed	5	8	5	8
6	35	Right	18	More than 1 year	Retired	5	8	8	9
7	34	Right	9	months	Unemployed	2	4	5	8
8	48	Right	14	More than 1 year	Retired	4	5	10	10
9	42	Left	7	More than 1 year	Retired	3	5	4	5/8
10	46	Right	10	More than 1 year	Retired	6	8	0	8
11	32	Right	7	More than 1 year	Employed	6	9	0	8
12	50	Left	10	More than 1 year	Retired	2	8	5/6	7/8
13	23	Right	10	More than 1 year	employed	3	6	4	7
14	65	Right	10	More than 1 year	Employed	5	5	0	5

Average pain measured as 0 = no pain; 10 = worst imaginable.

Difficulties with personal care and housework measured as 0 = no difficulty; 10 = impossible to do.

All participants completed all tests. Due to excessive shoulder pain, participant 8 only completed one trial (with and without orthoses) of the box and block test and the coin test for the dominant and non-dominant sides. Participant 9 only completed one trial on the coin test (with and without orthoses). In this instance, the task was so difficult for the participant that she felt she could not manage an additional trial. In these cases, data from the single trial that were performed were included in the analysis.

fNIRS data for the tBBT for participant 11 were found to be faulty due to a technical error. This data was removed from the analysis. tBBT data for the right DLPFC for participant 10 were also removed due to technical errors in the data.

### Targeted b and block test

Significant differences were observed in the time taken to complete the tBBT test using both the dominant and non-dominant hand (p < 0.01)(see Table 2). In both cases, participants completed the test in a shorter period of time when they used their orthoses. The effect size of the difference was large on the dominant side and moderate on the non-dominant side.

**Table 2.**
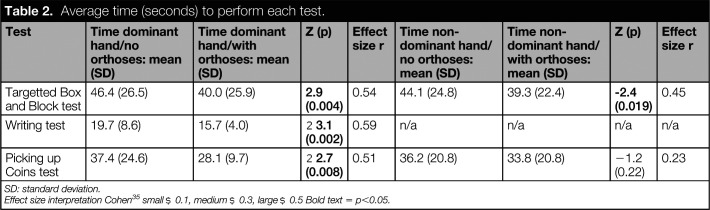
Average time (seconds) to perform each test.

Test	Time dominant hand/no orthoses: mean (SD)	Time dominant hand/with orthoses: mean (SD)	Z (p)	Effect size r	Time non-dominant hand/no orthoses: mean (SD)	Time non-dominant hand/with orthoses: mean (SD)	Z (p)	Effect size r
Targetted Box and Block test	46.4 (26.5)	40.0 (25.9)	**2.9 (0.004)**	0.54	44.1 (24.8)	39.3 (22.4)	**-2.4 (0.019)**	0.45
Writing test	19.7 (8.6)	15.7 (4.0)	**−3.1 (0.002)**	0.59	n/a	n/a	n/a	n/a
Picking up Coins test	37.4 (24.6)	28.1 (9.7)	**−2.7 (0.008)**	0.51	36.2 (20.8)	33.8 (20.8)	−1.2 (0.22)	0.23

SD: standard deviation.

Effect size interpretation Cohen^35^ small ≥ 0.1, medium ≥ 0.3, large ≥ 0.5 Bold text = p<0.05.

**Figure 4.**
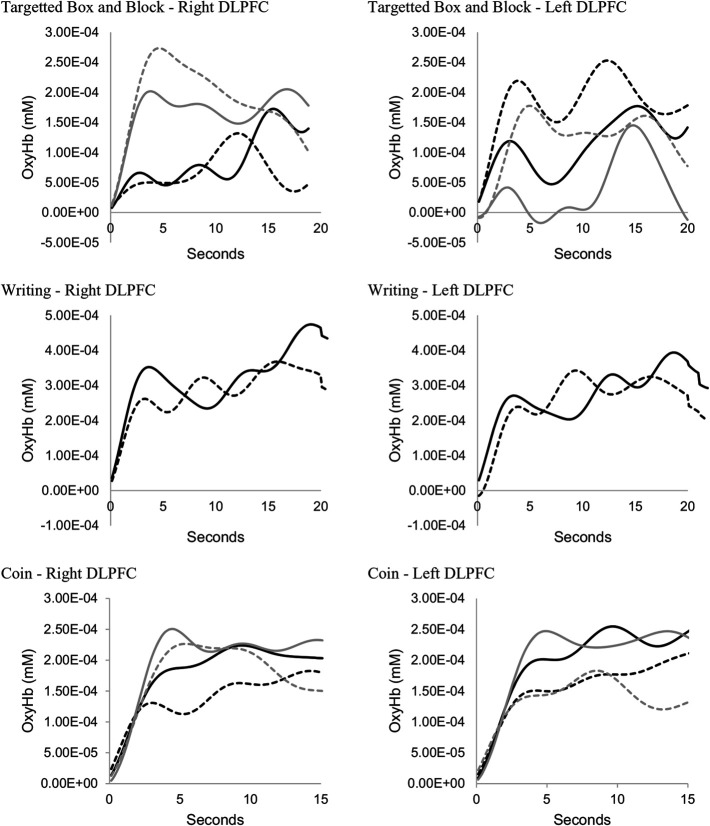
Average OxyHb over time (all participants) solid line = no orthoses, dotted line = orthoses, black = dominant hand, grey = non-dominant hand.

Figure 4 presents fNIRS data for the left and right DLPFC, averaged across all participants as a function of time. No difference was observed in fNIRS data between the orthosis and no orthosis conditions (Table 3). Nor was there any significant correlation between time to complete the test and cortical brain activity (Table 4).

**Table 3.**
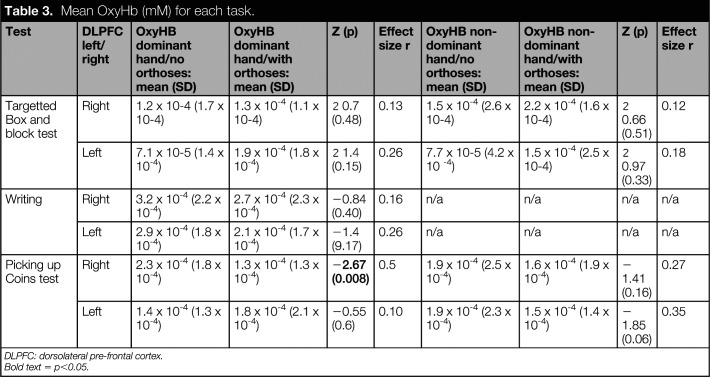
Mean OxyHb (mM) for each task.

Test	DLPFC left/right	OxyHB dominant hand/no orthoses: mean (SD)	OxyHB dominant hand/with orthoses: mean (SD)	Z (p)	Effect size r	OxyHB non-dominant hand/no orthoses: mean (SD)	OxyHB non-dominant hand/with orthoses: mean (SD)	Z (p)	Effect size r
Targetted Box and block test	Right	1.2 x 10-4 (1.7 x 10-4)	1.3 x 10^-4^ (1.1 x 10-4)	−0.7 (0.48)	0.13	1.5 x 10^-4^ (2.6 x 10-4)	2.2 x 10^-4^ (1.6 x 10-4)	−0.66 (0.51)	0.12
	Left	7.1 x 10-5 (1.4 x 10^-4^)	1.9 x 10^-4^ (1.8 x 10^-4^)	−1.4 (0.15)	0.26	7.7 x 10-5 (4.2 x 10 ^-4^)	1.5 x 10^-4^ (2.5 x 10-4)	−0.97 (0.33)	0.18
Writing	Right	3.2 x 10^-4^ (2.2 x 10^-4^)	2.7 x 10^-4^ (2.3 x 10^-4^)	−0.84 (0.40)	0.16	n/a	n/a	n/a	n/a
	Left	2.9 x 10^-4^ (1.8 x 10^-4^)	2.1 x 10^-4^ (1.7 x 10^-4^)	−1.4 (9.17)	0.26	n/a	n/a	n/a	n/a
Picking up Coins test	Right	2.3 x 10^-4^ (1.8 x 10^-4^)	1.3 x 10^-4^ (1.3 x 10^-4^)	−**2.67 (0.008)**	0.5	1.9 x 10^-4^ (2.5 x 10^-4^)	1.6 x 10^-4^ (1.9 x 10^-4^)	−1.41 (0.16)	0.27
	Left	1.4 x 10^-4^ (1.3 x 10^-4^)	1.8 x 10^-4^ (2.1 x 10^-4^)	−0.55 (0.6)	0.10	1.9 x 10^-4^ (2.3 x 10^-4^)	1.5 x 10^-4^ (1.4 x 10^-4^)	−1.85 (0.06)	0.35

DLPFC: dorsolateral pre-frontal cortex.

Bold text = p<0.05.

**Table 4.**
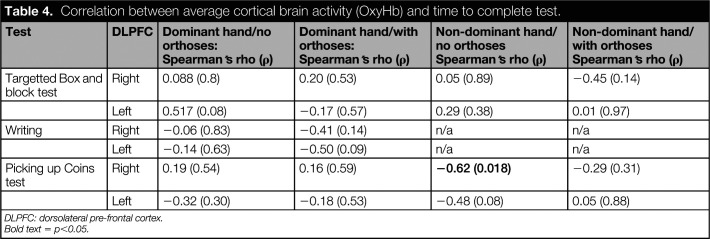
Correlation between average cortical brain activity (OxyHb) and time to complete test.

Test	DLPFC	Dominant hand/no orthoses: Spearman’s rho (ρ)	Dominant hand/with orthoses: Spearman’s rho (ρ)	Non-dominant hand/no orthoses Spearman’s rho (ρ)	Non-dominant hand/with orthoses Spearman’s rho (ρ)
Targetted Box and block test	Right	0.088 (0.8)	0.20 (0.53)	0.05 (0.89)	−0.45 (0.14)
	Left	0.517 (0.08)	−0.17 (0.57)	0.29 (0.38)	0.01 (0.97)
Writing	Right	−0.06 (0.83)	−0.41 (0.14)	n/a	n/a
	Left	−0.14 (0.63)	−0.50 (0.09)	n/a	n/a
Picking up Coins test	Right	0.19 (0.54)	0.16 (0.59)	**−0.62 (0.018)**	−0.29 (0.31)
	Left	−0.32 (0.30)	−0.18 (0.53)	−0.48 (0.08)	0.05 (0.88)

DLPFC: dorsolateral pre-frontal cortex.

Bold text = p<0.05.

### Writing test

Time to complete the writing test was significantly shorter when participants used their finger orthoses (p < 0.01)(Table 2). The effect size of the difference was found to be large. No significant difference was observed in fNIRS data averaged across each trial (Table 3) and no significant correlations were observed (Table 4). The graphical representation of cortical activity over time (Figure 4) suggests little difference between the orthosis and noorthosis conditions.

### Picking up coins test

Participants required a significantly shorter period of time to complete the picking up coins test with their dominant hand than they did when they used their finger orthoses (p < 0.01). The effect size of this difference was found to be large. No difference was observed between the orthosis and no orthosis condition on the non-dominant side (Table 2).

A significant difference was observed when comparing fNIRS data in the right DLPFC when participants were required to pick up the coins with their dominant hand (p < 0.01) (Table 3; Figure 4). No other significant differences were observed when comparing fNIRS data between the orthosis and no orthosis conditions. A significant negative correlation was, however, observed between time to perform the test with the non-dominant hand and cortical brain activity in the right DLPFC (rho = -0.62; p = 0.018).

## Discussion

Investigations into the use of finger orthoses to prevent undesired movement in individuals with hypermobility disorders has not previously been addressed in research literature. The present study was designed to investigate the effects of finger orthosis use on functional performance and attentional demand in individuals with JHS, hEDS or cEDS. Results confirmed our hypothesis that use of finger orthoses would significantly reduce time to perform functional hand tests, however, an expected reduction in cortical brain activity in the prefrontal cortex was only observed in one test (picking up coins with dominant hand).

Functional performance was measured by the time to complete three different tests using three different grips. Results for the dominant hand were consistent across all three tests and demonstrated a significant reduction in time to complete the test. Effect sizes were large for all three tests performed with the dominant hand. The writing test was not conducted with the non-dominant hand, however, a significant reduction in time to perform the tBBT test was observed (medium effect size). No difference was recorded for the picking up coins test performed with the non-dominant hand.

Based upon results of previous studies using fNIRS,^43,44^ and in light of the observed improvements in functional performance, we expected to see a reduction in cortical brain activity in the region of the pre-frontal cortex when participants performed hand function tests with their finger orthoses. This was the case when participants were picking up coins with their dominant hand, however, no differences were observed in the remaining tests. We can only speculate as to why this was the case. Given that activity in the pre-frontal cortex is associated with task difficulty,^45^ one potential explanation is that the tasks were not challenging enough and subsequently required minimal attentional demand both with and without orthoses. In future research, it would be interesting to select tests which require the same grip but are increasingly difficult to perform. One could then correlate task difficulty with cortical brain activity. The type of grip required to perform each test may also have influenced results. The picking up coins test required that participants use a precision grip while the tBBT test required a less precise pinch grip that would have been less challenging for participants with finger impairments.

It is possible that an attentional bias towards pain may have affected our results. Previous work has demonstrated an interaction between pain and cognitive load in the medial prefrontal cortex.^46,47^ We subsequently suggest that future studies include a detailed description of pain experienced with and without the finger orthoses and to include an analysis of cortical brain activity for individuals experiencing high levels of pain against those with low or no pain.

There are several limitations that must be acknowledged in relation to this study. The first being a relatively small sample which leads to greater sampling variability. There was also substantial variation in the participants’ age and gross motor function which would also increase variability. Unfortunately, we did not collect data on finger range of motion for participants in this study. Potential variability between participants is a limitation of this study that should be accounted for in future work. It must also be recognised that participants used only one type of orthosis and the effects of orthosis design on outcomes has not been addressed in this research. It should also be noted that all testing was completed on one day, which could have resulted in fatigue and increased pain towards the end of testing. While we randomised the order of testing with and without orthoses to control for this, there is a possibility that results may have been affected. The single testing session used in this study also meant that the no orthosis condition and the orthosis condition were assessed on the same day, and we were unable to explore any effects over time.

## Conclusion

Results of this study suggest that finger orthoses may have a positive effect on hand function in individuals with JHS, hEDS or cEDS. While improved benefits in cognitive load, measured as cortical brain activity in the prefrontal cortex, were only observed in the task requiring a fine precision grip with the dominant hand. We suggest that combined results provide sufficient evidence to pursue further research into the potential benefits of finger orthosis prescription in this clinical population.

## Supplemental Material

No supplemental digital content is available in this article.

## References

[1] Tinkle B Castori M Berglund B, et al. Hypermobile Ehlers-Danlos syndrome (a.k.a. Ehlers-Danlos syndrome Type III and Ehlers-Danlos syndrome hypermobility type): clinical description and natural history. Am J Med Genet C Semin Med Genet 2017; 175: 48–69. 28145611 10.1002/ajmg.c.31538

[2] Malfait F Francomano C Byers P, et al. The 2017 international classification of the Ehlers-Danlos syndromes. Am J Med Genet C Semin Med Genet 2017; 175: 8–26. 28306229 10.1002/ajmg.c.31552

[3] US. National Library of Medicine. Ehlers-Danlos syndrome, 2020, https://ghr.nlm.nih.gov/condition/ehlers-danlos-syndrome#statistics (accessed 15 March 2020).

[4] Castori M Dordoni C Valiante M, et al. Nosology and inheritance pattern(s) of joint hypermobility syndrome and Ehlers-Danlos syndrome, hypermobility type: a study of intrafamilial and interfamilial variability in 23 Italian pedigrees. Am J Med Genet A 2014; 164A: 3010–3020. 25338840 10.1002/ajmg.a.36805

[5] Krahe AM Adams RD and Nicholson LL. Features that exacerbate fatigue severity in joint hypermobility syndrome/Ehlers–Danlos syndrome – hypermobility type. Disabil Rehabil 2018; 40: 1989–1996. 28482708 10.1080/09638288.2017.1323022

[6] Hope L Juul-Kristensen B LÃvaas H, et al. Subjective health complaints and illness perception amongst adults with joint hypermobility syndrome/ehlers-danlos syndrome-hypermobilitytype – a cross-sectional study. Disabil Rehabil 2019; 41: 333–340. 29041819 10.1080/09638288.2017.1390695

[7] Saetre E and Eik H. Flexible bodies-restricted lives: a qualitative exploratory study of embodiment in living with joint hypermobility syndrome/ehlers-danlos syndrome, hypermobility type. Musculoskeletal Care 2019; 17: 241–248. 31347287 10.1002/msc.1407

[8] Castori M Morlino S Celletti C, et al. Re-writing the natural history of pain and related symptoms in the joint hypermobility syndrome/Ehlers-Danlos syndrome, hypermobility type. Am J Med Genet A 2013; 161A: 2989–3004. 24254847 10.1002/ajmg.a.36315

[9] Adib N Davies K Grahame R, et al. Joint hypermobility syndrome in childhood: a not so benign multisystem disorder? Rheumatology 2005; 44: 744–750. 15728418 10.1093/rheumatology/keh557

[10] Terry RH Palmer ST Rimes KA, et al. Living with joint hypermobility syndrome: patient experiences of diagnosis, referral and self-care. Fam Pract 2015; 32: 354–358. 25911504 10.1093/fampra/cmv026 PMC4445137

[11] Castori M and Hakim A. Contemporary approach to joint hypermobility and related disorders. Curr Opin Pediatr 2017; 29: 640–649. 28906340 10.1097/MOP.0000000000000541

[12] Smith TO Jerman E Easton V, et al. Do people with benign joint hypermobility syndrome (BJHS) have reduced joint proprioception? A systematic review and meta-analysis. Rheumatol Int 2013; 33: 2709–2716. 23728275 10.1007/s00296-013-2790-4

[13] Scheper MC Nicholson LL Adams RD, et al. The natural history of children with joint hypermobility syndrome and Ehlers-Danlos hypermobility type: a longitudinal cohort study. Rheumatology 2017; 56: 2073–2083. 28431150 10.1093/rheumatology/kex148

[14] Galli M Rigoldi C Celletti C, et al. Postural analysis in time and frequency domains in patients with Ehlers-Danlos syndrome. Res Dev Disabil 2010; 32: 322–325. 21071172 10.1016/j.ridd.2010.10.009

[15] Hernandez ME Holtzer R Chaparro G, et al. Brain activation changes during locomotion in middle-aged to older adults with multiple sclerosis. J Neurol Sci 2016; 370: 277–283. 27772776 10.1016/j.jns.2016.10.002

[16] Kurz MJ Wilson TW and Arpin DJ. An fNIRS exploratory investigation of the cortical activity during gait in children with spastic diplegic cerebral palsy. Brain Dev 2014; 36: 870–877. 24508407 10.1016/j.braindev.2014.01.003 PMC4122656

[17] Stuart S Belluscio V Quinn JF, et al. Pre-frontal cortical activity during walking and turning is reliable and differentiates across young, older adults and people with Parkinson’s disease. Front Neurol 2019; 10: 536–536. 31191434 10.3389/fneur.2019.00536 PMC6540937

[18] Möller S Rusaw D Hagberg K, et al. Reduced cortical brain activity with the use of microprocessor-controlled prosthetic knees during walking. Prosthet Orthot Int 2019; 43: 257–265. 30375285 10.1177/0309364618805260

[19] Kwon G Kim M-Y Lim S, et al. Frontoparietal EEG alpha-phase synchrony reflects differential attentional demands during word recall and oculomotor dual-tasks. Neuroreport 2015; 26: 1161–1167. 26559729 10.1097/WNR.0000000000000494

[20] Izzetoglu K Bunce S Izzetoglu M, et al. Functional near-infrared neuroimaging. Conf Proc IEEE Eng Med Biol Soc 2004; 7: 5333–5336. 10.1109/IEMBS.2004.1404489 17271546

[21] Smith TO Bacon H Jerman E, et al. Physiotherapy and occupational therapy interventions for people with benign joint hypermobility syndrome: a systematic review of clinical trials. Disabil Rehabil 2014; 36: 797–803. 23889528 10.3109/09638288.2013.819388

[22] Bale P Easton V Bacon H, et al. The effectiveness of a multidisciplinary intervention strategy for the treatment of symptomatic joint hypermobility in childhood: a randomised, single centre parallel group trial (The Bendy Study). Pediatr Rheumatol Online J 2019; 17: 2. 30621718 10.1186/s12969-018-0298-x PMC6325876

[23] Barati H Zarezadeh A MacDermid JC, et al. The immediate sensorimotor effects of elbow orthoses in patients with lateral elbow tendinopathy: a prospective crossover study. J Shoulder Elbow Surg 2019; 28: e10–e17. 30551783 10.1016/j.jse.2018.08.042

[24] Frohlich L Wesley A Wallen M, et al. Effects of neoprene wrist/hand splints on handwriting for students with joint hypermobility syndrome: a single system design study. Phys Occup Ther Pediatr 2012; 32: 243–255. 21992010 10.3109/01942638.2011.622035

[25] von Elm E Altman DG Egger M, et al. The strengthening the reporting of observational studies in epidemiology (STROBE) statement: guidelines for reporting observational studies. Int J Surg 2014; 12: 1495–1499. 25046131 10.1016/j.ijsu.2014.07.013

[26] Castori M Camerota F Celletti C, et al. Ehlers–Danlos syndrome hypermobility type and the excess of affected females: possible mechanisms and perspectives. Am J Med Genet A 2010; 152A: 2406–2408. 20684008 10.1002/ajmg.a.33585

[27] Wilcox T and Biondi M. fNIRS in the developmental sciences. Wiley Interdiscip Rev Cogn Sci 2015; 6: 263–283. 26263229 10.1002/wcs.1343 PMC4979552

[28] Irani F Platek SM Bunce S, et al. Functional near infrared spectroscopy (fNIRS): an emerging neuroimaging technology with important applications for the study of brain disorders. Clin Neuropsychol 2007; 21: 9–37. 17366276 10.1080/13854040600910018

[29] Herold F Wiegel P Scholkmann F, et al. Applications of functional near-infrared spectroscopy (fNIRS) neuroimaging in exercise–cognition science: a systematic, methodology-focused review. J Clin Med 2018; 7:466. 10.3390/jcm7120466 PMC6306799 30469482

[30] Desrosiers J Bravo G Hébert R, et al. Validation of the Box and Block Test as a measure of dexterity of elderly people: reliability, validity, and norms studies. Arch Phys Med Rehabil 1994; 75: 751–755. 8024419

[31] Kontson K Marcus I Myklebust B, et al. Targeted box and blocks test: normative data and comparison to standard tests. PLoS ONE 2017; 12: e0177965. 28542374 10.1371/journal.pone.0177965 PMC5438168

[32] Jebsen R Taylor N Trieschmann R, et al. An objective and standardized test of hand function. Arch Phys Med Rehabil 1969; 50: 311–319. 5788487

[33] Singh H Dias J and Thompson JR. Timed Sollerman hand function test for analysis of hand function in normal volunteers. J Hand Surg Eur Vol 2015; 40: 298–309. 24565857 10.1177/1753193414523246

[34] Zhu Y Rodriguez-Paras C Rhee J, et al. Methodological approaches and recommendations for functional near-infrared spectroscopy applications in HF/E research. Hum Factors 2020; 62: 613–642. 31107601 10.1177/0018720819845275

[35] Zimeo Morais GA Balardin JB and Sato JR. fNIRS optodes’ location decider (fOLD): a toolbox for probe arrangement guided by brain regions-of-interest. Sci Report 2018; 8: 3341–3341. 10.1038/s41598-018-21716-z PMC5820343 29463928

[36] Fishburn FA Norr ME Medvedev AV, et al. Sensitivity of fNIRS to cognitive state and load. Front Hum Neurosci 2014; 8: 76. 24600374 10.3389/fnhum.2014.00076 PMC3930096

[37] Fraser SA Dupuy O Pouliot P, et al. Comparable cerebral oxygenation patterns in younger and older adults during dual-task walking with increasing load. Front Aging Neurosci 2016; 8: 240. 27812334 10.3389/fnagi.2016.00240 PMC5071361

[38] Pheifer M Scholkmann F and Labruyére R. Signal processing in functional near-infrared spectroscopy (fNIRS): methodological differences lead to different statistical results. Front Aging Neurosci 2018; 11: 1–11. 10.3389/fnhum.2017.00641 PMC5766679 29358912

[39] Piper SK Krueger A Koch SP, et al. A wearable multi-channel fNIRS system for brain imaging in freely moving subjects. Neuroimage 2014; 15: 64–71. 10.1016/j.neuroimage.2013.06.062 PMC3859838 23810973

[40] de Lima-Pardini AC Zimeo Morais GA Balardin JB, et al. Measuring cortical motor hemodynamics during assisted stepping: an fNIRS feasibility study of using a walker. Gait Posture 2017; 56: 112–118. 28544947 10.1016/j.gaitpost.2017.05.018

[41] Field A. Discovering statistics using IBM SPSS statistics. 4th ed. London: SAGE, 2013.

[42] Cohen J. Statistical power analysis for the behavioral sciences. 2nd ed. New York: Lawrence Erlbaum Associates, 1988.

[43] Ramstrand N Rusaw DF and Möller SF. Transitioning to a microprocessor-controlled prosthetic knee: executive functioning during single and dual-task gait. Prosthet Orthot Int 2019; 44: 27–35. 31826702 10.1177/0309364619892773

[44] Hawkins KA Fox EJ Daly JJ, et al. Prefrontal over-activation during walking in people with mobility deficits: interpretation and functional implications. Hum Mov Sci 2018; 59: 46–55. 29604488 10.1016/j.humov.2018.03.010 PMC5988641

[45] Vassena E Gerrits R Demanet J, et al. Anticipation of a mentally effortful task recruits dorsolateral prefrontal cortex: an fNIRS validation study. Neuropsychologia 2019; 123: 106–115. 29705065 10.1016/j.neuropsychologia.2018.04.033

[46] Wiech K Lin CS Brodersen KH, et al. Anterior insula integrates information about salience into perceptual decisions about pain. J Neurosci 2010; 30: 16324–16331. 21123578 10.1523/JNEUROSCI.2087-10.2010 PMC6634837

[47] Wiech K Seymour B Kalisch R, et al. Modulation of pain processing in hyperalgesia by cognitive demand. Neuroimage 2005; 27: 59–69. 15978845 10.1016/j.neuroimage.2005.03.044

